# Is there any relation between anthropometric indices and decrease in seminal parameters?

**DOI:** 10.1590/S1679-45082014AO2781

**Published:** 2014

**Authors:** Juliana Christofolini, Raphael Augusto Saab de Almeida Barros, Milton Ghirelli-Filho, Denise Maria Christofolini, Bianca Bianco, Caio Parente Barbosa

**Affiliations:** 1 Faculdade de Medicina do ABC, Santo André, SP, Brazil

**Keywords:** Adiposity, Male infertility, Body mass index, Semen, Reproduction

## Abstract

**Objective:**

To investigate the influence of anthropometric indices on seminal parameters.

**Methods:**

Men who underwent treatment for conjugal infertility during the period of October, 2011, to March, 2012, were randomly selected. Patients with any prior diseases related to sperm alterations were excluded. Patients were submitted to an anthropometric evaluation to obtain body mass index, and the seminal analysis was made through a spermogram. Two anthropometric methods of classification were used: body mass index (normal and altered) and abdominal circumference (<94cm and >94cm). Data were analyzed by statistical tests.

**Results:**

The group with the altered body mass index presented lower volumes of ejaculated volume and a larger percentage of patients with abdominal circumference <94cm presented with progressive forms of spermatozoa below reference values. However, in the statistical tests, there was no significant difference.

**Conclusion:**

No significant difference was found in the sperm quality relative to the body mass index or abdominal circumference.

## RESUMO

**Objetivo:**

Investigar a influência dos índices antropométricos em parâmetros seminais

**Métodos:**

Foram selecionados de maneira aleatória homens que procuraram tratamento para infertilidade conjugal durante o período de outubro de 2011 até março de 2012. Foram excluídos os pacientes com quaisquer doenças relacionadas a alterações espermáticas prévias. Os pacientes passaram por avaliação antropométrica para obtenção do índice de massa corporal, e a análise seminal foi feita por meio de espermograma. Foram utilizados dois métodos de classificação antropométrica: índice de massa corporal (normal e alterado) e circunferência abdominal (<94cm e >94cm). Os dados foram analisados por meio de teste estáticos.

**Resultados:**

O grupo com índice de massa corporal alterado apresentou menores valores de volume de ejaculado, e uma fração maior de pacientes com circunferência abdominal <94cm apresentou formas progressivas de espermatozoide abaixo dos valores de referência. No entanto, nos testes estatísticos, não houve diferença significativa.

**Conclusão:**

Não foi encontrada diferença significativa na qualidade espermática em relação ao índice de massa corporal ou à circunferência abdominal.

## INTRODUCTION

Obesity is a severe health problem observed in the entire world.^(1)^ Approximately 1.6 billion adults (aged over 15 years) are classified as being overweight (body mass index – BMI – between 25 and 30kg/m^2^) and 400 million as obese (BMI ≥30kg/m^2^).^(1)^ The weight of Brazilians has also been increasing over the last years. Data from the Research on Family Budgets (POF, acronym in Portuguese for *Pesquisa de Orçamentos Familiares)* applied by the Brazilian Institute of Geography and Statistics (IBGE) in a partnership with the Ministry of Health showed that during the period between 2008 and 2009, excess weight already affected half of Brazilians, regardless of gender.^(2)^ Obesity is an important health risk factor and is associated with high morbidity and mortality, especially due to cardiovascular diseases^(3,4)^ and diabetes.^(5)^ It is also associated with other diseases, such as cancer^(6)^ and non-communicable chronic diseases, such as osteoarthritis,^(7)^ liver and gall bladder diseases,^(8)^ sleep apnea,^(9)^ depression,^(10)^ and infertility.^(11)^

The consequences of obesity in female fertility have been widely studied, but studies in the male population are less frequent.^(12)^ It has been postulated that obese men have an increased risk of erectile dysfunction.^(13)^ It has also been proposed that overweight and obesity in men can lead to a drop in levels of sex hormone-binding globulin (SHBG), increasing the levels of estradiol and altering secretion of gonadotropins.^(14,15)^

A significant reduction of testosterone levels relative to the serum levels of estradiol was observed in men with excess weight or obesity (BMI>25kg/m^2^) when compared to the serum levels of men with a lower BMI^(15)^. This variation would be associated with the accumulation of adipose tissue,^(15,16)^ and the consequent hormone variation that can lead to oligozoospermia. More recently, it has also been described that overweight and obesity are related to lower levels of inhibin B,^(17,18)^ a marker of Sertoli cell function and spermatogenesis. Additionally, some studies have investigated if the excess weight affects the integrity of DNA of the sperm,^(19)^ as this is an independent marker of seminal quality^(20)^ that predicts fertility.^(21,22)^

Also reported was a higher prevalence of oligozoospermia in men with overweight/obesity relative to men with an appropriate weight. Nevertheless, no relation was found between the increase in BMI and the percentage of mobile sperm.^(23)^

Other authors reported a negative correlation between obesity and various seminal parameters in the general population. Nonetheless, there is controversy as to the extent of this relation.^(23-25)^

## OBJECTIVE

To investigate the influence of body mass index and abdominal circumference on seminal parameters.

## METHODS

Male patients were invited to participate in the study among couples with reproductive difficulties that searched/underwent treatment for infertility at the *Instituto Ideia Fértil de Saúde Reprodutiva do Centro de Reprodução Humana e Genética da Faculdade de Medicina do ABC*, recruited in a consecutive manner during the period between October, 2011 and March, 2012. Patients with varicocele, cryptorchidism, hypospadia, trauma, vasectomy reversal, and chromosome alterations and/ or micro deletion of the Y chromosome were excluded from the study.

Clinical and anthropometric data were only collected after presenting the objectives of the study and signing of the Informed Consent Form, approved by the Research Ethics Committee of the *Instituto de Saúde e Bem Estar da Mulher* (ISBEM).

Therefore, 118 men were selected for evaluation of BMI and abdominal circumference. The mean age of the men was 35.59±7.47 years, and for their partners, 34.03±5.39 years. The mean time of infertility of the couples was 3.38±3 years.

Ninety-three individuals (76.85%) declared not having any comorbidity and also no use of any type of medication. As to genital diseases or malformations, 105 (86.77%) declared not having any disease/malformation. Of the patients who had any complaint, 5 cited sexually transmissible diseases (4.14%) and the other 11 patients mentioned other diseases and malformations, such as inguinal hernias, hydrocele, and phimosis (9.09%).

BMI was calculated as per the Quetelet formula, dividing the weight in kilograms by the squared height in meters: BMI=weight inkg/(height in m)^2^.

To verify the influence of the anthropometric parameters on the variables of seminal analysis, participants were separated as per their nutritional status by their BMI, creating a control group (18<BMI≤25) and a study group (BMI>25).

Taking into consideration that the BMl may have a bias relative to the patients that present with a high percentage of lean mass, who can be classified with BMI>25, the abdominal circumference measurement was also used as a separation criterion of the participants in a second analysis. Therefore, two groups were obtained, one as control (abdominal circumference <94cm) and one study group (abdominal circumference ≥94cm). The abdominal circumference was measured with an inelastic measuring tape at the level of the umbilical scar with volunteers in the orthostatic position.

For these groups, the spermogram was performed and evaluated in a timely fashion and according to the recommendations of the World Health Organization (WHO) in 2010. The cutoff values for sperm normality adopted for the variables were: volume (V) >1.5mL; total concentration (TC) >39 million; initial concentration (IC) >15 million; progressive (PR) >32% or progressive + non-progressive forms (NP)=40%.

Data analysis was done with the Statistical Package for Social Sciences (SPSS) program, version 16.0. Student's *t* test was used to compare among the variables and the χ^2^ test and Kruskal-Wallis's non-parametric test for qualitative variables with a statistical significance value established at 5% or p<0.05.

## RESULTS

The distribution of the number of patients according to the BMI is shown on table 1. The mean weight of patients was 87.0±19.29kg, while the mean height was 1.75±0.074m.

**Table 1:** Distribution of the number of patients according to the body mass index

BMI	Number of patients (%)
<25	31 (26.2)
25≤BMI<30	56 (47.7)
30≤BMI<35	21 (17.7)
35≤BMI<40	5 (4.2)
≥40	5 (4.2)
Total	118 (100)

BMI: body mass index.

The abdominal circumference had a mean value of 96.42 and standard deviation of ±13.99cm. Distribution of the number of patients, according to classes of abdominal circumference is demonstrated on table 2.

**Table 2:** Distribution of the number of patients according to abdominal circumference

AC (cm)	Number of patients (%)
<94	54 (45.7)
≥94 and<102	30 (25.4)
≥102	34 (28.8)
Total	118 (100)

AC: abdominal circumference.

In the seminal analysis, the variables observed were V, TC, IC, and final concentration (FC), as well as the mobile and immobile forms of sperm.

Comparisons of the results of the seminal analysis among the groups of BMI and abdominal circumference are on table 3. The qualitative analysis between the groups showed no statistical difference in the results found, and the results of p value for the V *versus* BMI, and V relative to abdominal circumference ratios were 0.462 and 0.548, respectively. For TC of sperm relative to the BMI, TC *versus* abdominal circumference, the p values were 0.932 and 0.378. As to IC and BMI, the IC and abdominal circumference were 0.297 and 0.833, respectively. The p values found for PR relative to the BMI, PR and the abdominal circumference were 0.875 and 0.169, respectively.

**Table 3:** Comparison of the seminal values of patients below reference values as per body mass index and abdominal circumference values

Variables	Patients with BMI>25	Patients with BMI ≤25	p value	Patients with AC>94	Patients com AC<94	p value
Volume <1.5mL	14.94% (n 13)	9.68% (n 3)	0.462	12.50% (n 8)	16.36% (n 9)	0.548
TC <39 million	21.84% (n 19)	22.58% (n 7)	0.932	18.75% (n 12)	25.45% (n 14)	0.378
IC <15 million	25.29% (n 22)	16.13% (5)	0.297	23.44 % (n 15)	21.82% (12)	0.833
PR initial <32%	14.94% (n 13)	16.13% (n 5)	0.875	10.94% (n 17)	20.00% (11)	0.169

TC: concentration total; IC: initial concentration; PR: progressive sperm forms; BMI: body mass index; AC: abdominal circumference.

## DISCUSSION

Male obesity has been the target of great discussion as to its impact on fertility, with much controversy in literature.

In the present paper, no statistical differences were found in the data analyzed between the BMI and abdominal circumference groups that would allow relating the drop in seminal quality of men with excess weight and/or obesity. Our results are similar to those of other larger studies, such as by Chavarro et al.,^(26)^ who studied 483 men, members of infertile couples, and also found no significant differences between obese and eutrophic men as to sperm concentration and their motility, despite finding a greater V of ejaculation in non-obese men. Similarly, Jessen et al.,^(18)^ in their study with 1558 Danish men, also found no significant differences between the total number of mobile sperm and the ejaculated V, but did show, however, a reduction in concentration and in total sperm count in the group of men with a lower BMI (<20kg/m^2^). On the other hand, other studies, showed that obesity leads to alterations in almost all seminal parameters, such as ejaculated V, and mobile and progressive forms of sperm.^(27,28)^

In the present study, we also found no significant difference when analyzing the abdominal circumference and the seminal parameters, contrasting with the findings of Fejes et al.,^(29)^ who noted in their study an association between increased adiposity and a drop in sperm motility. Nevertheless, we cannot disregard the fact that the size of our sample influenced our results. Corroborating our findings, Strain et al.^(30)^ found no alterations in the V ejaculated and in motility of the sperm, verifying as well that the total count of sperm and libido were not altered with increased obesity, suggesting that this picture of hypogonadotropic hypogonadism is mild.

Although our data support the statement that obesity does not lead to considerable changes in seminal parameters, there seem to be alterations in the fecundity of obese men. In our study, 87% of the participants were overweight or obese, while in another study,^(26)^ also performed in an infertility treatment clinic, 75% of the men were above ideal weight. Contrary to the still inconsistent findings regarding the influence of the BMI on the seminal parameters, there is a relation between excess weight and hormone parameters.^(24,26,31-34)^ Adding these findings to hormone alterations that demonstrate dysfunction of the seminiferous tubules and hypogonadotropic hypogonadism, we can support the hypothesis that obesity is a risk factor for a drop in fecundity and that, along with other mild risk factors, such as decreased libido and/or changes in sexual performance, may lead to infertility.^(24)^

## CONCLUSION

The present study found no statistical difference between the body mass index and abdominal circumference and the drop in seminal quality of men with excess weight and/or obesity.
